# Modelling of Friction Phenomena Existed in Drawbead in Sheet Metal Forming

**DOI:** 10.3390/ma14195887

**Published:** 2021-10-08

**Authors:** Tomasz Trzepieciński, Andrzej Kubit, Romuald Fejkiel, Łukasz Chodoła, Daniel Ficek, Ireneusz Szczęsny

**Affiliations:** 1 Department of Manufacturing and Production Engineering, Rzeszow University of Technology, al. Powst. Warszawy 8, 35-959 Rzeszów, Poland; akubit@prz.edu.pl; 2 Department of Mechanics and Machine Building, Carpatian State School in Krosno, ul. Żwirki i Wigury 9A, 38-400 Krosno, Poland; romuald.fejkiel@kpu.krosno.pl; 3 Department of Integrated Design and Tribology Systems, Faculty of Mechanics and Technology, Rzeszow University of Technology, ul. Kwiatkowskiego 4, 37-450 Stalowa Wola, Poland; l.chodola@prz.edu.pl; 4 Department of Aerospace Engineering, Faculty of Mechanical Engineering and Aeronautics, Rzeszow University of Technology, al. Powst. Warszawy 8, 35-959 Rzeszów, Poland; ficekd@prz.edu.pl (D.F.); rm@prz.edu.pl (I.S.)

**Keywords:** drawbead, coefficient of friction, friction, sheet metal forming, steel sheets

## Abstract

The article presents the results of friction tests of a 0.8 mm-thick DC04 deep-drawing quality steel sheet. A special friction simulator was used in the tests, reflecting friction conditions occurring while pulling a sheet strip through a drawbead in sheet metal forming. The variable parameters in the experimental tests were as follows: surface roughness of countersamples, lubrication conditions, sample orientation in relation to the sheet rolling direction as well as the sample width and height of the drawbead. Due to many factors that affect the value of the coefficient of friction coefficient, artificial neural networks (ANNs) were used to build and analyse the friction model. Four training algorithms were used to train the ANNs: back propagation, conjugate gradients, quasi-Newton and Levenberg–Marquardt. It was found that for all analysed friction conditions and sheet strip widths, increasing the drawbead height increases the COF value. The chlorine-based Heavy Draw 1150 compound provides a more effective friction reduction compared to a LAN-46 machine oil.

## 1. Introduction

In order to obtain the required quality of the drawpiece in the sheet metal forming (SMF) process, it is necessary to properly select the conditions of the sheet forming process, in which technological aspects should be considered, taking into account the stress and deformation states occurring in the sheet, as well as the frictional conditions between the pressed sheet and the tool [1,2,3]. The phenomenon of friction in sheet metal forming processes is complex and is not a static phenomenon, but is subject to significant fluctuations depending on the forming conditions [4,5]. A complete description of the frictional conditions during stamping is very complicated and requires taking into account the properties of the frictional pair of materials, their surface characteristics such as texture and roughness, mechanical properties of materials, as well as the quantity and properties of the lubricant, and finally various kinematic and dynamic process conditions [6,7,8]. The selection of appropriate friction conditions is extremely important, not only to ensure the required quality of the pressed pieces, but also to minimize the wear of the forming tools [8,9,10].

Due to the high pressures that occur on contact surfaces, the friction phenomenon in plastic working processes differs significantly from the friction occurring in machine parts and has a direct impact on the distribution of stresses and strains in the formed material [11,12]. The frictional conditions also affect the value of the force necessary for deformation, as well as the value of pressure on the working surfaces of the tools, which, in turn, affects the aspects of tool wear [13,14]. The most common in this type of processes is adhesive wear, which also includes scuffing [15], another frequently occurring form of wear is abrasive wear [16,17]. Friction can cause the phenomena of the shaped material sticking to the working surfaces of the tool, which also accelerates their wear and significantly affects the surface quality of the products [18].

In a typical sheet metal drawing process, the surface of the sheet is lubricated. The lubricant changes the contact conditions between the tool and the sheet surface. According to Gierzyńska [19], in the process of sheet deformation, a lubricant reduces unit pressure and the coefficient of friction (COF), changes the flow character of the deformed metal and its topography, and improves the quality of the product surface. The appropriate shape of the surface of tools, creating conditions for the formation of the so-called oil pockets, significantly improves lubrication efficiency. Experimental research on this subject was conducted, among others, by the authors of the works [20,21,22]. The deformation of the geometric structure resulting from pressure distribution, with a different sample orientation, in different ways favours the formation of oil pockets as micro-areas of hydrodynamic lubrication [23]. 

In SMF, drawbeads are one way to regulate and control the material flow during the forming process in order to prevent wrinkling [24]. Drawbeads impede the material flow of the sheet metal, but also change mechanical properties due to the work hardening phenomenon. Over the years, many researchers analysed the effect of drawbeads on material formability in SMF. Samuel [25] investigated the behaviour of metal flow which passes through different shapes of drawbeads. He concluded that the drawbead restraining force is affected greatly by friction conditions and bead geometry. Leocata et al. [26] carried out the strip tensile test to analyse the restraining force for different pressure zones. It was found that an increased surface roughness leads to a decreased sensitivity of friction to variations in the amount of lubricant. Zhongqin et al. [27] studied the effect of the geometrical shape and dimensions of the drawbead on the drawbead restraining force (DBRF) and material flow. They concluded that the experimental approach to obtain proper drawbead geometry is not only expensive, but also requires much effort and time. Lee et al. [28] developed a numerical method to predict the DBRF. Comparisons between measurements and calculations for SPCC steel showed that hardening behaviour precisely predicts the DBRF. Murali et al. [29] numerically analysed a circular and rectangular drawbead position on the die surface and their effect on the thickness distribution over the formed cup. They found that circular drawbeads are preferred since the thickness reduction is lesser than when rectangular drawbeads are used. Bassoli et al. [30] developed a handy simulator to measure the DBRF during the deep drawing of 6014-T4 aluminium alloy metal sheet. The results show that some geometries of the drawbead enabled nearly invariant dynamic behaviour, in that the restraining force was almost insensitive to changes in friction conditions. Schmid et al. [31] modified a strip drawing test to observe the material behaviour of DC04 steel while passing a drawbead. An overall increase in hardness while passing the sheet through a drawbead was pointed out. Hardness increases due to an overlying tensile load and it can be seen that passing a drawbead leads to an increase in material hardness.

Artificial neural networks (ANNs) are one of the most frequently used methods of solving problems in friction testing and analysis of wear. The prospects for the use of artificial intelligence in tribology have been discussed in the paper of Rosenkranz et al. [32]. Lemu et al. [33] applied radial basis function (RBF) ANNs to create a mathematical model of friction behaviour based on the results of the strip drawing test. They found that the back propagation algorithm is the most efficient learning algorithm. Trzepieciński and Szpunar [34] built an empirical model of friction of Ti-6Al-4V titanium sheets with the use of RBF ANNs. It was concluded that an increase in the number of radial neurons in the hidden layer caused an increase in the value of the determination coefficient and a reduction in the standard deviation ratio. Otero et al. [35] investigated the use of ANNs for the prediction of the COF in elastohydrodynamic lubrication point contacts. It was highlighted that special care is needed when using ANN models for prediction since they are accompanied with the loss of relevant information, or intermediate results of interest. Boidi et al. [36] employed the RBF for predicting the COF in lubricated contacts with textured and porous surfaces. Prajapati and Tiwari [37] demonstrated the potential of ANN in the prediction of roughness parameters, COF and wear coefficients in pin-on-disk tribotesting. A very good agreement of the results suggested that a well-trained ANN is capable of predicting the parameters in the wear process. Argatov et al. [38] applied a multilayer perceptron for data-driven modelling of the wear coefficient in sliding wear under constant testing conditions. They provided examples of the use of the approach developed on the basis of the experimental data published recently. In the last two decades in particular, the areas of successful incorporation of ANNs have been constantly expanding in tribological research and cover such diverse applications as erosion of polymers [39], brake performance [40,41], tool wear [42], wear of journal bearings [43], wheel and rail wear [44], the microabrasion-corrosion process [45] and wear of polymer composites [46,47]. Neural networks optimised by genetic algorithms can be also used for the design of lubricant formulations in tribology [48,49]. Humelnicu et al. [50] also analysed the use of ANNs to design lubricants with significantly lower COFs. Predictions of COFs for optimised mixtures of vegetable oils aligned well with the experimental results. Examples of ANN applications in tribological studies were discussed by Argatov [51]. Sha and Edwards [52] recommended suitable guidelines for the proper handling of ANNs to reveal their potential for effective modelling and analysis of tribological problems. The potential applications of ANNs in the field of tribology have been reviewed by Frangu and Ripa [53].

Most studies in the literature only determine the influence of selected geometric parameters of the friction process arising in a drawbead during SMF. Moreover, the vast majority of results presented in the literature are based on the pulling of a strip of sheet metal of the same width. On the basis of preliminary tests, it was found that the width of the sheet significantly affects the deformation of the sheet while passing the sheet strip through a drawbead, and thus, the value of the coefficient of friction. Therefore, it was decided to prepare and present the results of extensive friction investigations in this paper. In experimental tests a friction simulator was used to determine the influence of lubrication conditions, the height of the drawbead as well as the sample width and its orientation on the value of the COF. Based on the results of experimental research, an analytical model of friction was built using artificial neural networks trained with various training algorithms. The influence of particular parameters of the friction test on the COF value was presented and discussed. Based on experimental results, the qualitative comparison of the ANN model was carried out.

## 2. Materials and Methods

### 2.1. Material

**Figure 1:**
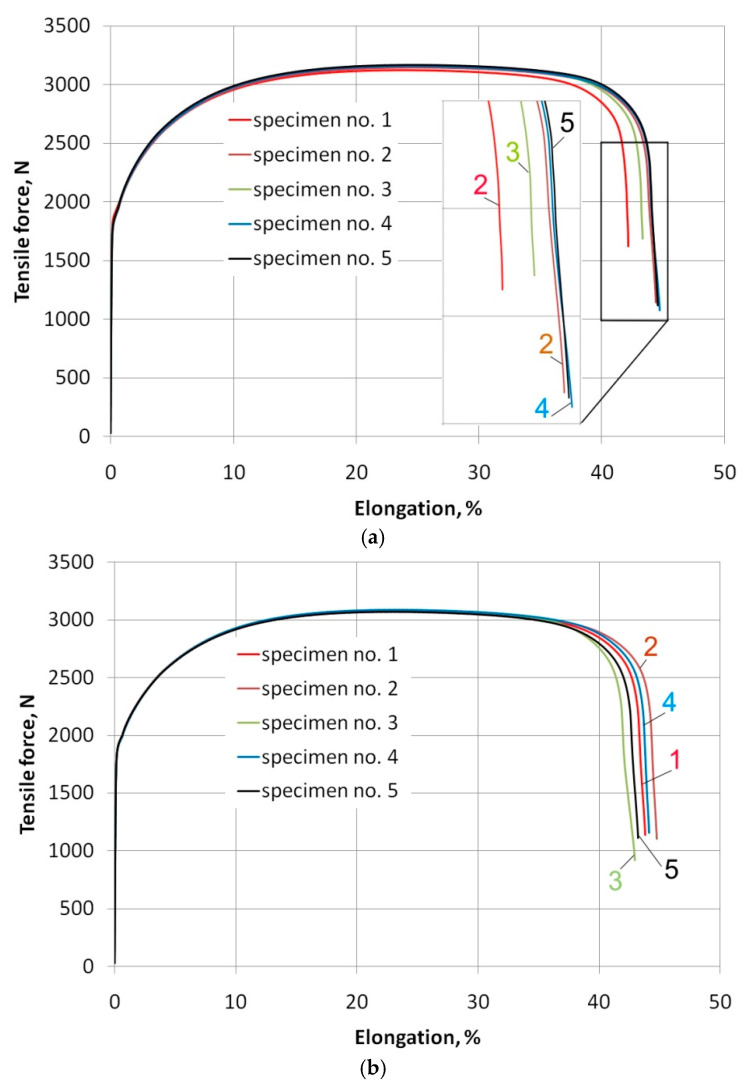
Load-elongation curves for specimens cut (**a**) parallel and (**b**) transversely to the sheet rolling direction.

As test material a 0.8 mm-thick cold rolled low carbon steel DC04 was used. The research material meets the requirements of EN 10130: 2009 [54]. This grade is suitable for high deformation requirements and is commonly used in the automotive industry. In order to determine basic mechanical properties, strength tests were carried out on the universal testing machine Zwick Roell Z030 according to the EN ISO 6892-1: 2016-09 [55] standard. In the experimental tests of friction, the strips cut along the rolling direction RD (α = 0°) and transversely to this direction (α = 90°) were tested. Therefore, uniaxial stretching tests were performed for these two directions. The mean values of basic material parameters (Table 1) were determined as the mean values of five measurements. Values of strain hardening parameters were determined by approximating the relationship true stress-true strain relationship obtained in the uniaxial tensile test using the Hollomon equation *σ* = *K*·*ε^n^*, where *σ* is true stress, *K* is the strengthening coefficient, *ε* is the true strain and *n* is the strain hardening exponent. Figure 1 shows load-elongation curves from a tensile test.

**Figure 2:**
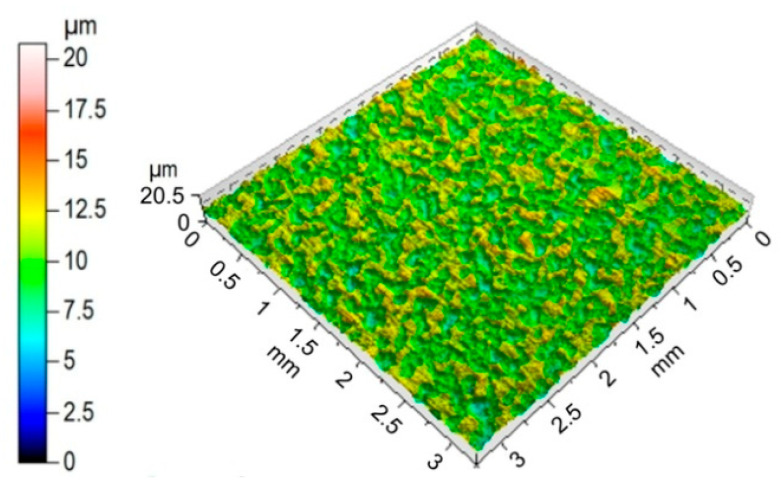
Topography of the DC04 steel sheet.

The surface roughness of the sheets tested was measured before the friction process with the Talysurf CCI Lite 3D scanning profilometer according to the EN ISO 25178-6 [56] international standard. The surface topography of the DC04 sheet metal and basic surface roughness parameters are shown in Figure 2 and Table 2, respectively.

### 2.2. Friction Test

The curvature of the metallic sheet passing through the drawbead model is changed several times; the sheet is alternately bent and straightened. Thus, the direction of the friction force changes along the curvature and does not coincide with the direction of the pulling force that is measured. The idea behind the method is to provide the ability to separate the deformation resistance of the sheet from the friction. In the DBT, the values of the pulling force and the clamping force are measured when pulling the strip over fixed and rotatable rollers.

**Figure 3:**
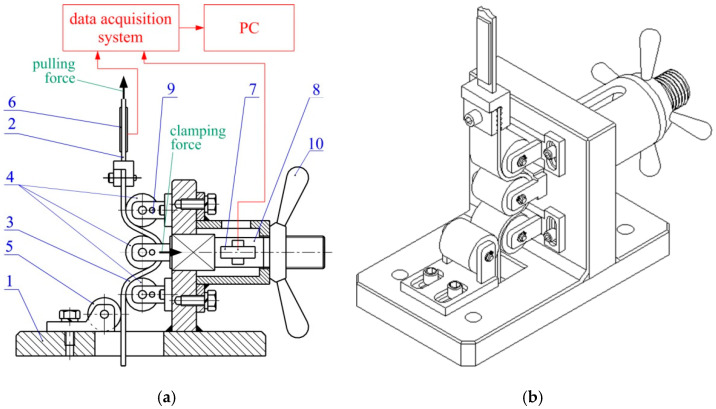
(**a**) diagram and (**b**) 3D model of the test device: 1—frame; 2—vertical tension member; 3—specimen; 4—working rolls; 5—supporting roll; 6,7—load cells; 8—horizontal tension member; 9—pin, 10—nut.

The value of the COF while passing the sheet strip through the drawbead was determined using a special tribological simulator (Figure 3). The tested strip of sheet metal is bent, unbent and reverse bent many times passing between rolls 1, 2 and 3. A symmetrical guidance of the sheet in relation to the middle roll 2 is provided by the supporting roller 4. It also prevents the sheet end from bending when it enters the working roller 3. The device was mounted on a Zwick/Roell Z100 testing machine (Zwick/Roell GmbH & Co. KG, Ulm, Germany. One end of the sheet strip was mounted in the upper gripper of the testing machine. The force values were recorded at a frequency of 50 Hz using the Lab View DAQ program integrated with the NI 9237 measurement card.

According to the idea of separating the sheet deformation resistance from friction resistance, two tests should be carried out: (i) with rotating rollers and (ii) with fixed rollers. The value of the friction coefficient is determined from the relationship [57]:

(1) $$\mu =\frac{F_{cn}-F_{co}}{F_{dn}}\frac{sin\theta }{2\theta }\;\;\;\;\;\;$$

where *F_co_* is the pulling force obtained with the freely rotating rolls, *F_cn_* is the pulling force obtained with the fixed rolls, *F_dn_* is the clamping force obtained with the fixed beads, *Θ* is the half contact angle of the strip over the middle roller.

**Figure 4:**
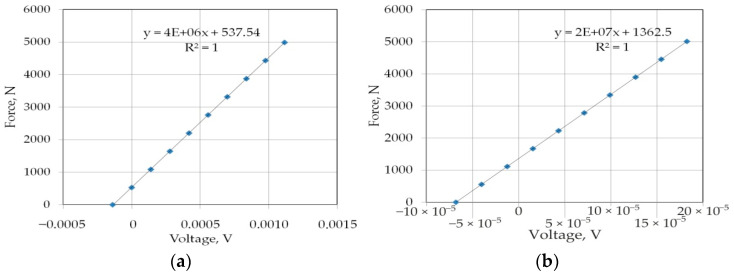
Calibration curves for strain gauge sensors measured for (**a**) pulling and (**b**) clamping forces.

The clamping force of the central roller *F_dn_* was measured using strain gauge 8 (Figure 3), and the pulling forces *F_co_* and *F_cn_* were measured using strain gauge 7 (Figure 3). The strain gauges were calibrated using a Zwick Roell Z100 professional uniaxial testing machine (load capacity F_nom_ = 100 kN, stiffness of the load frame 500 kN/mm). Force measurement accuracy with a load cell was equal to Class 1 for loads from 0.4 to 100%F_nom_ and Class 0.5 for loads from 2 to 100%F_nom_ (the accuracy is 0.5% of the reading under full load) [58]. The effect of calibrating the strain gauge sensors are graphs showing the dependence of the recorded tension force values and excitation voltage [59]. The force measured by strain gauges shows linear agreement with the indications of the Zwick Roell Z100 professional testing machine. The excitation voltages were registered with an accuracy of 0.00001 V using the NI 9237 strain/bridge input module and transferred into forces according to the regression functions shown in Figure 4.

Angle *Θ* can be determined on the basis of the arrangement of rolls simulating the drawbead (Figure 4):

(2) $$\theta =\frac{\pi }{2}-2arc\;tg\;\left(\frac{2R+g+h}{2R+c}\right)$$

where *g* is the sheet thickness, *R* is the radius of roller, *h* is the drawbead height and *c* is the side clearance.

**Figure 5:**
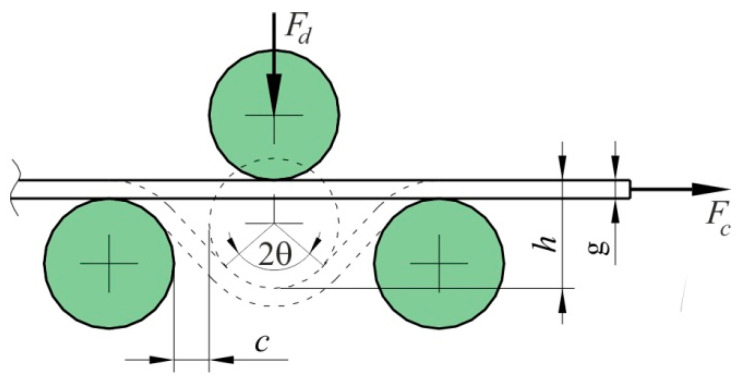
Geometric parameters of the drawbead.

During the tests, an adequate side clearance *c* (Figure 5) should be ensured between rolls in order to prevent the sheet strip from blocking between rollers [60]. At high angles of contact, the sample may even break. On the other hand, applying an excessive side clearance between rolls can adversely change the way the sheet is deformed [61]. In preliminary tests, the value of the side clearance *c* was experimentally determined as 1.5 of the sheet thickness.

The tests were carried out under dry friction conditions and lubrication with LAN-46 machine oil and chlorine-based Heavy Draw (HD) 1150 synthetic stamping and drawing compound. The viscosity of the HD 1150 compound is *η* = 1157 mm^2^·s^−1^, while the viscosity of the LAN-46 oil is *η* = 43.9 mm^2^·s^−1^. The strip specimens were lubricated using a teflon shaft [11,62]. The amount of lubricant applied to each of the two surfaces of the samples was 2 g/m^2^ [11,62]. During the test the lubricant is squeezed out between the countersample surface and the sheet surface. Moreover, the thickness of oil is not uniform along the total area of contact. The load is carried on roughness asperities where the lubricant thickness is equal to zero, and in the rest of the area, the thickness of the lubricant layer depends on the volume of valleys in the surface topography.

The rest of the test conditions are as follows:

- Surface roughness of countersamples Ra 0.32, 0.63 and 1.25 μm;
- Specimen orientations α = 0° and α = 90°;
- Specimen widths *w*: 7, 14 and 20 mm;
- Drawbead heights *h*: 6, 12 and 18 mm.

During sheet metal forming the hardness and strength of the tool material is many times greater that the hardness and strength of the workpiece material. Due to this fact and the fact that the values of specific forces registered during the test do not exceed 1500 N, it was assumed that the elastic deformation of the tool steel is negligibly small. Taking account of the analysis of recent developments and trends in friction testing for conventional sheet metal forming [63], the majority of researchers testing friction in sheet metal forming focus on producing a device with appropriately high stiffness. The elastic strains of the countersamples themselves are not considered in the macroscopic analysis of the friction forces [64,65,66,67,68].

### 2.3. Surface Characterization

The surfaces of the sheets after friction tests were examined using Phenom ProX scanning electron microscope (Nanoscience Instruments, Phoenix, AZ, USA).

### 2.4. Artificial Neural Networks

Due to a large number of parameters influencing the value of the COF determined in the drawbead friction test, it is difficult to determine the synergistic relationship between input parameters and the value of COF. ANNs, by simulating the flow of information in the human brain, are able to model a problem of any complexity [69,70]. Therefore, this article uses ANNs to determine the model of variation in COF during the drawbead test. To ensure the quality of the neural network, it is necessary to provide a training data set. The following set of variables was selected as input signals to the network:

- Average surface roughness of countersamples;
- Lubrication conditions;
- Orientation of the sheet metal strip with respect to the sheet rolling direction;
- Drawbead height;
- Sample width.

The aim of the training process is to select weights in individual neurons in order to minimise a global error of the ANN. As a result of the learning process, ANNs can acquire the ability to predict output signals based on the sequence of input signals and the corresponding output signals. The task of the learning algorithm is to select the weight values and threshold values of all neurons in such a way as to ensure the minimisation of error in network operation. The learning algorithm, starting from the initial random system of weights and threshold values, modifies these values in a manner that tries to reach the overall minimum. All the training datasets are presented to the network during each iteration. The error value is determined from the difference between the values of the output signals and the standards.

The set of data (input signals and the corresponding COF value) obtained from experimental friction tests was used to train the ANN. Basic learning algorithms were used to train the network: back propagation (BP), conjugate gradients (CG), quasi-Newton (q-N) and Levenberg–Marquardt (LM). As a result of the learning process, the trained neural network acquires the ability to predict the value of the output signal based on the sequence of input signals and the corresponding output signals presented during the learning process. The task of the training algorithm is to select the threshold values and weights of neurons in order to minimise the global error of the ANN.

**Figure 6:**
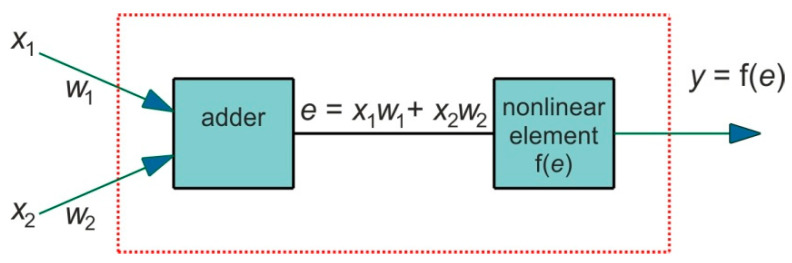
Structure of an individual neuron.

Each neuron consists of two modules. The first one adds the products of the weighting factors and the input signals. In the second module, the output signal from the first module is processed by the neuron activation function. The signal *e* (Figure 6) is the output signal of the adder, while *y* = f(*e*) is the output signal of the non-linear element. The signal *y* is also the output signal of the neuron. Training the network with the back propagation algorithm is an iterative process. In each iteration, the neuron weight factors are modified using new data from the training dataset. Each learning step (epoch) begins with forcing all input signals from the training set. After this stage, the values of the output signals are determined for each neuron in each network layer. 

The Levenberg–Marquardt algorithm is a fast-convergent algorithm. Its computational complexity is not very large and its implementation is simple [71]. The work principle of the LM algorithm is based on the least-squares method [72]. The LM algorithm, also known as the damped least-squares method, works without computing the exact value of the Hessian matrix of the error function. The LM regularization method consists of replacing the Hessian matrix with its approximation based on gradient calculations with a properly selected regularization factor. The algorithm of the LM method approximates the Hessian of the error function by means of an appropriate transformation of the residual matrix and Jacobian. Jacobians (derivatives of the outputs with respect to the network weights and with respect to the inputs) are used to determine the sensitivity of the network outputs [73].

In the quasi-Newton method, the Hessian of the minimized error function is approximated by analysing successive gradient vectors. The variable-metric method assumes that the error function can be approximated by a quadratic function in the neighbourhood of the local optimum. The fact that the Hessian satisfies the condition of positive definiteness at each step of the ANN’s training makes the qN method one of the best methods for optimizing multivariable functions. Due to the high computational complexity related to the necessity to calculate n^2^ elements of Hessian, this method is recommended for relatively not very complex neural networks.

The conjugate gradient algorithm is an iterative method of ANN training. In the CG algorithm [74,75], the direct use of the Hessian matrix for the construction of a new direction of the minimum search is abandoned. This method is based on the assumption that in order to ensure the correct training process, the direction of the search for the minimum error function should be coupled to the previous gradient value [76].

Among all training pairs (162 input signals and the corresponding output signal), 10% [33,77] were randomly selected and included in the validation set. The moment the RMS error value of the validation set no longer decreases was adopted as the criterion for completing the network training process [33,77]:

(3) $$RMS=\sqrt{\frac{\sum_{i=1}^{N}{\left(z_{i}-y_{i}\right)}^{2}}{N}}$$

where *z_i_* is the expected signal of the output neuron for the *i* -th pattern, *y_i_* is the signal of the output neuron for the *i* -th pattern, *N* is the number of vectors in the training set.

The normalization of values of all input data to the range [*N_min_* (0), *N_max_* (1)] was performed using the min-max method:

(4) $$D^{\prime }=\frac{\left(D-min\right)}{max-min}\left(N_{max}-N_{min}\right)+N_{min}$$

where *D* is the value of the variable subjected to normalization and (min, max) is the range in which the original data are contained.

In the Statistica Neural Networks (SNNs) program, a number of ANNs models were built for a different number of neurons. The number of neurons in the input layer was determined by the number of input parameters. At the output of the network there was one neuron responsible for the COF value.

## 3. Results and Discussion

### 3.1. The Effect of Drawbead Height

**Figure 7:**
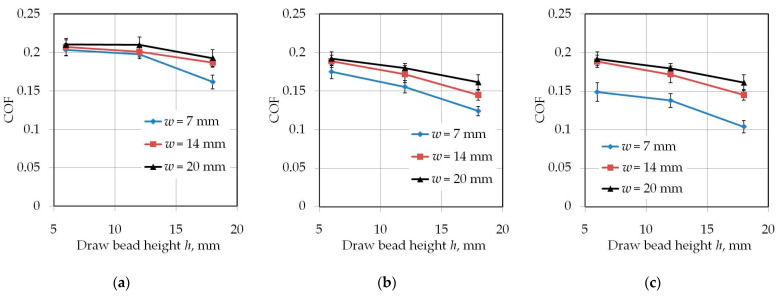
The effect of the drawbead height on the value of COF for specimens cut along the RD (orientation 0°): (**a**) dry friction, (**b**) LAN-46 oil lubrication, (**c**) HD 1150 compound lubrication.

**Figure 8:**
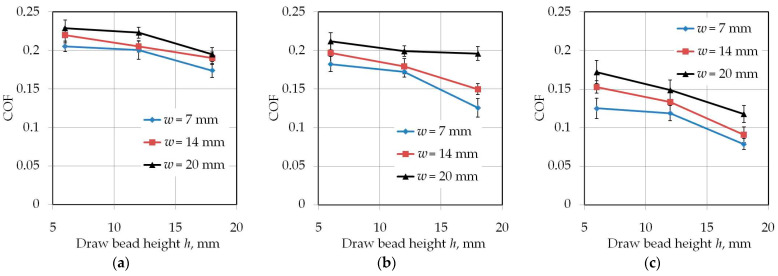
The effect of the drawbead height on the value of COF for specimens cut transverse to the RD (orientation 90°): (**a**) dry friction, (**b**) LAN-46 oil lubrication, (**c**) HD 1150 compound lubrication.

**Figure 9:**
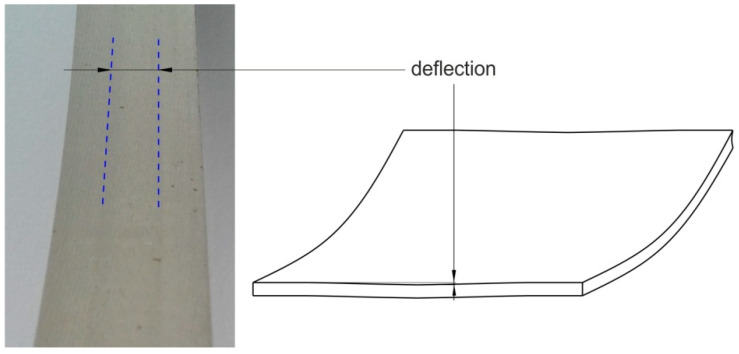
Deflection of the sheet strip (*w* = 20 mm) after passing the drawbead.

Increasing the drawbead height increases the value of the friction coefficient (Figure 7 and Figure 8). Such a dependence occurs for all friction conditions and the widths of the sheet strip. First of all, it should be explained why different sheet widths were used in the tests. The shape of the cross-section of the bent sheet strip depends on the ratio of the strip width to its thickness. The larger this parameter, the more the sheet has a concave shape, which was observed in previous research [78] while bending the same sheet as used in research presented in this manuscript. The sheet metal passed through the drawbead takes on a concave shape in the middle part (Figure 9), which limits the real contact area. So, the width of the sheet strip determines the different manner of deformation, and thus, the different real contact area of the sheet with the countersamples. The results presented in Figure 7 clearly confirm that the width of the sheet strip tested determines the change in COF. The above conclusions can be extended to the sample orientation of 90° (Figure 8).

Proof that the contact area has an influence on COF in the drawbead test is the work of Nanayakkara et al. [57]. They conducted experiments with different drawbead penetrations *h*. The change in drawbead penetration *h* changes the contact area between the sheet and the countersamples. COF was not constant for a wide range of drawbead heights. In a similar manner to changing the contact area by changing the bead penetration, the width of the sample also plays an important role. The method of deformation of the sheet depending on its width changes the proportions between the pulling and clamping forces measured during the test and, consequently, the COF value. This phenomenon has been studied numerically in a recent paper of the authors [79].

### 3.2. Effect of Countersample Roughness

**Figure 10:**
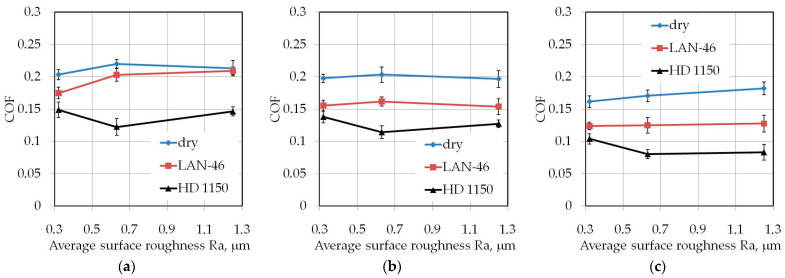
Effect of countersample roughness on the value of COF for specimens with a width of *w* = 7 mm cut along the rolling direction (orientation 0°): (**a**) *h* = 6 mm, (**b**) *h* = 12 mm, (**c**) *h* = 18 mm.

The influence of average surface roughness Ra of countersamples on the value of COF depends on the width of the test sample. Lubrication of the sheet surface using the HD 1150 compound while testing samples with a width of 7 mm (Figure 10) produced a favourable effect at Ra = 0.63 μm. At this roughness, the lubricant provides the lowest COF value. The beneficial effect of the volume of the lubricant pockets and the high viscosity of the HD 1150 lubricant is clearly visible.

**Figure 11:**
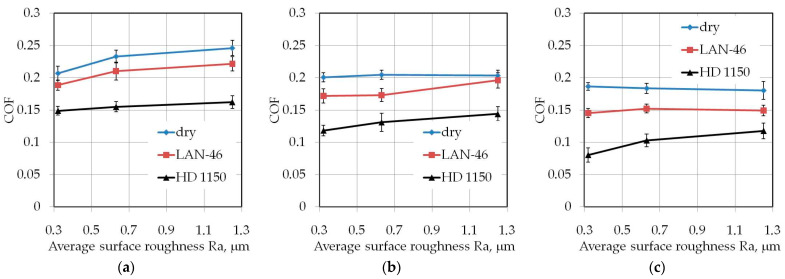
Effect of countersample roughness on the value of COF for specimens with a width of *w* = 14 mm cut along the rolling direction (orientation 0°): (**a**) *h* = 6 mm, (**b**) *h* = 12 mm, (**c**) *h* = 18 mm.

Undeniably, the HD 1150 compound provides a more effective reduction in COF values compared to LAN-46 machine oil. When pulling strips twice the width (Figure 11), the HD 1150 compound provided the greatest reduction in COF at an average surface roughness of countersamples Ra = 0.32 μm. Increasing the roughness of the countersamples also reduces lubrication efficiency. This conclusion can also be made on machine oil, however only for a drawbead height of *h* = 6 mm (Figure 11a) and *h* = 12 mm (Figure 11b).

**Figure 12:**
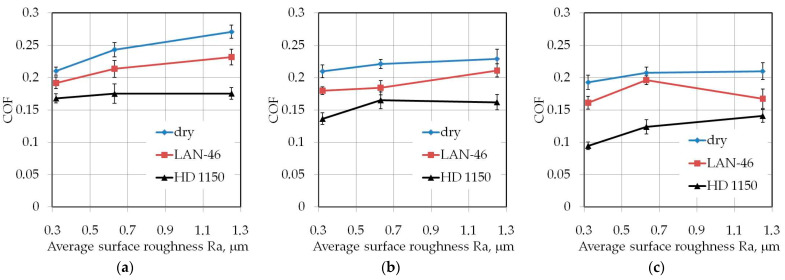
Effect of countersample roughness on the value of COF for specimens with a width of *w* = 20 mm cut along the rolling direction (orientation 0°): (**a**) *h* = 6 mm, (**b**) *h* = 12 mm, (**c**) *h* = 18 mm.

The tests with the drawbead height of *h* = 18 mm (Figure 11c) did not show a significant effect of the drawbead height on the lubrication efficiency of LAN-46 machine oil. As in the dry friction conditions (Figure 11b,c), the COF value is similar in terms of the analysed countersample roughnesses. A clear tendency to increase the COF when increasing the countersamples’ surface roughness was observed when testing samples with the largest width *w* = 20 mm (Figure 12) only under dry friction conditions in the whole range of the analysed drawbead heights.

### 3.3. The Effect of the Sample Orientation

**Figure 13:**
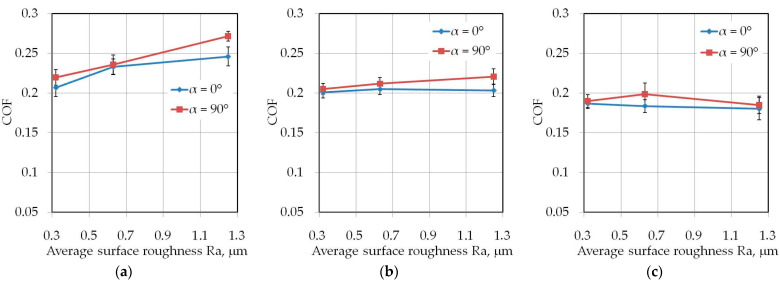
The effect of the sample orientation on the value of COF for specimens with a width of 14 mm tested at dry friction conditions: (**a**) *h* = 6 mm, (**b**) *h* = 12 mm, (**c**) *h* = 18 mm.

**Figure 14:**
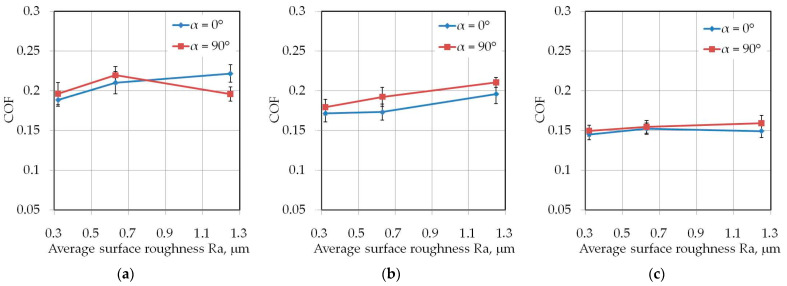
The effect of the sample orientation on the value of COF for specimens with a width of 14 mm tested at LAN-46 oil lubrication conditions: (**a**) *h* = 6 mm, (**b**) *h* = 12 mm, (**c**) *h* = 18 mm.

**Figure 15:**
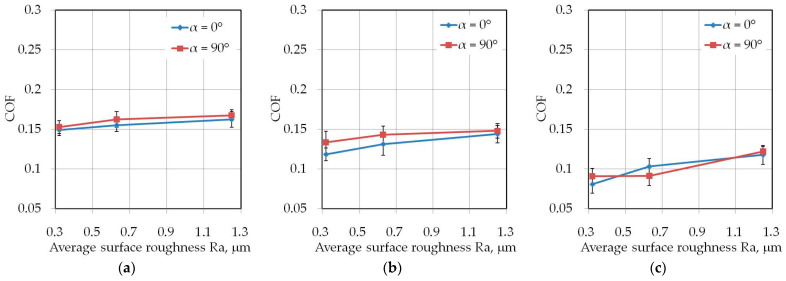
The effect of the sample orientation on the value of COF for specimens with a width of 14 mm tested at HD 1150 compound lubrication conditions: (**a**) *h* = 6 mm, (**b**) *h* = 12 mm, (**c**) *h* = 18 mm.

Although the sheets tested exhibit anisotropic material properties in relation to yield stress and strain hardening parameters (Table 1), the effect of specimen orientation on the value of COF is quite complicated. Values of the COF for the two analysed sample orientations did not differ by more than 0.025 (Figure 13, Figure 14 and Figure 15). However, in most of the analysed cases, the COF value was higher for the sample orientation 90°. As mentioned in Section 2.1, the sheets tested were fabricated in a cold rolling process. It is well known that this process induces directional microstructure in the material. The grains are elongated in shape in the direction of the rolling process. Resistance to multiple bending, unbending and reverse bending of the sheet while passing through the drawbead is lower for samples oriented transversely to the sheet rolling direction. Moreover, flat specimens cut along the rolling direction show higher values of spring back while bending than samples cut transversely to the sheet rolling direction [80].

### 3.4. The Effect of Friction Conditions

**Figure 16:**
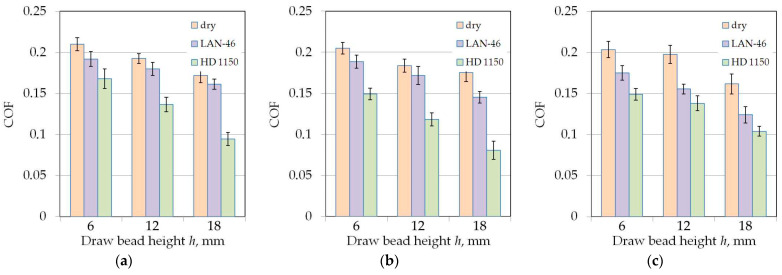
The effect of friction conditions on the value of COF for specimens cut along the sheet rolling direction (orientation 0°) tested using countersamples with a surface roughness of Ra = 0.32 μm: (**a**) *w* = 7 mm, (**b**) *w* = 14 mm, (**c**) *w* = 20 mm.

**Figure 17:**
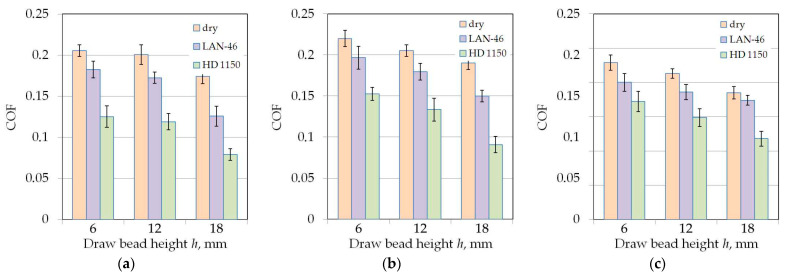
The effect of friction conditions on the value of COF for specimens cut transverse to the sheet rolling direction (orientation 90°) tested using countersamples with a surface roughness of Ra = 0.32 μm: (**a**) *w* = 7 mm, (**b**) *w* = 14 mm, (**c**) *w* = 20 mm.

The HD 1150 compound reduced the value of the COF to the greatest extent (Figure 16 and Figure 17). This lubricant most significantly reduced COF during tests with the highest analysed drawbead height *h* = 18 mm (Figure 16c or Figure 17c). With an increase in the drawbead height, lubrication efficiency decreases. For a drawbead height of *h* = 6 mm, lubrication with machine oil and the HD 1150 compound reduces the COF value by 11.19–27.75% and 26.74–54.65%, respectively. For a drawbead height of *h* = 18 mm, lubrication with machine oil and the HD 1150 compound reduces the COF value by 5.94–12.67% and 19.97–44.97%, respectively. The relations presented in Figure 14 and Figure 15 refer to the roughness of countersamples Ra = 0.32 μm. However, similar qualitative conclusions can be drawn for the rest of the analysed surface roughness of countersamples.

### 3.5. Friction Mechanisms

In SMF processes, there is a movement of a plasticized metal of relatively low hardness over the surface of the tool with a higher hardness. The forming load acts only on the roughness asperities which results in a higher degree of surface flattening and thus, a higher fraction of real contact area. Changes in the properties of the surface layer of the deformed material are related to the change of friction conditions. The basic friction mechanisms observed during the tests were flattening, roughening and adhesion. Adhesive wear is a form of wear characterized by high wear rates and a high unstable coefficient of friction. Frictional joints are quickly destroyed as a result of adhesive wear, and in extreme cases, sliding movement may be impossible as a result of seizure.

**Figure 18:**
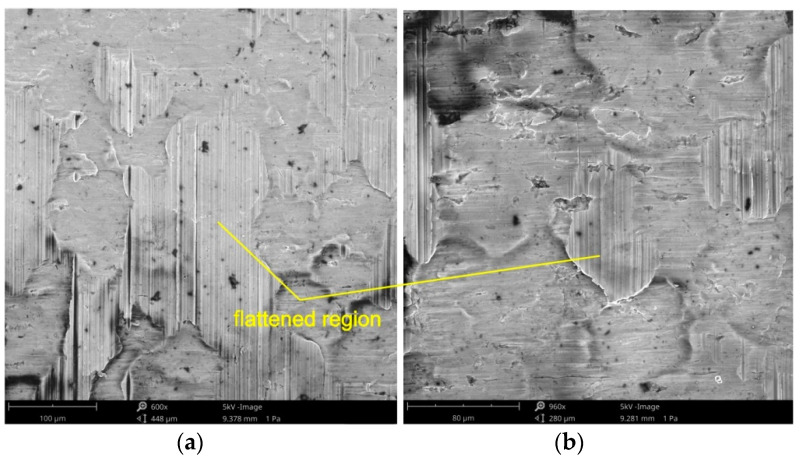
The specimen surfaces tested in lubricated conditions using LAN-46 machine oil (Ra = 0.32 μm, *h* = 6 mm, *w* = 14 mm, orientation 90°) at different magnifications: (**a**) ×600 and (**b**) ×960.

**Figure 19:**
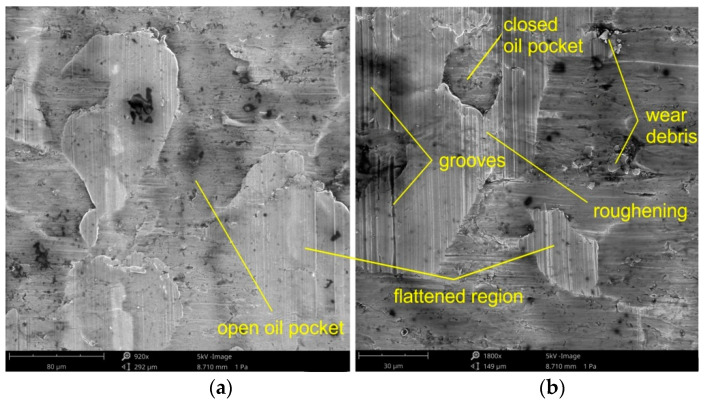
The specimen surfaces tested in dry friction (Ra = 0.32 μm, *h* = 6 mm, *w* = 14 mm, orientation 90°) at different magnifications: (**a**) ×920 and (**b**) ×1800.

The three dominating flattening mechanisms during SMF are: flattening due to normal loading, (ii) flattening due to normal load and (iii) flattening due to sliding. Flattening manifests itself in regions with flattened asperities of surface roughness (Figure 18 and Figure 19). Flattening increases the real area of contact, resulting in a higher COF value. Flattened regions carry the entire load of the tool and are surrounded by valleys, where the surface remains in an “as received” state. These areas constitute a reservoir of lubricant known as "oil pockets". The lubricant existing in closed oil pockets (Figure 19b) is pressurized while forming under load and increases its hydrostatic pressure [81]. Open oil pockets (Figure 19a) are connected to the edge of the surface and do not contain lubricant, which escapes when increasing the normal load.

**Figure 20:**
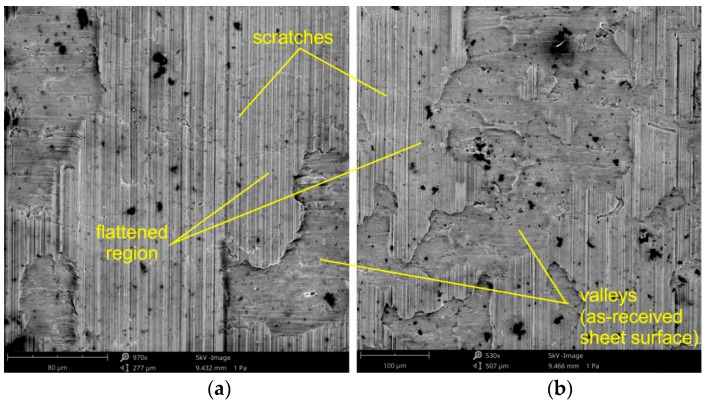
The specimen surfaces tested in lubricated conditions using LAN-46 machine oil (Ra = 0.63 μm, *h* = 12 mm, *w* = 14 mm, orientation 90°) at different magnifications: (**a**) ×970 and (**b**) ×530.

**Figure 21:**
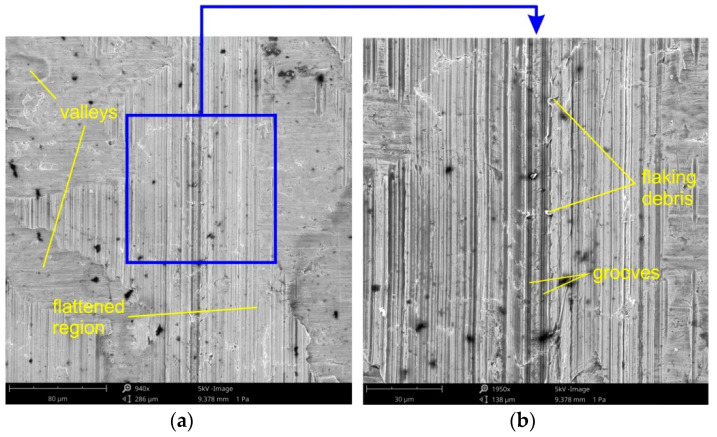
The specimen surfaces tested in dry friction (Ra = 0.63 μm, *h* = 12 mm, *w* = 14 mm, orientation 90°) at different magnifications: (**a**) ×940 and (**b**) ×1950.

**Figure 22:**
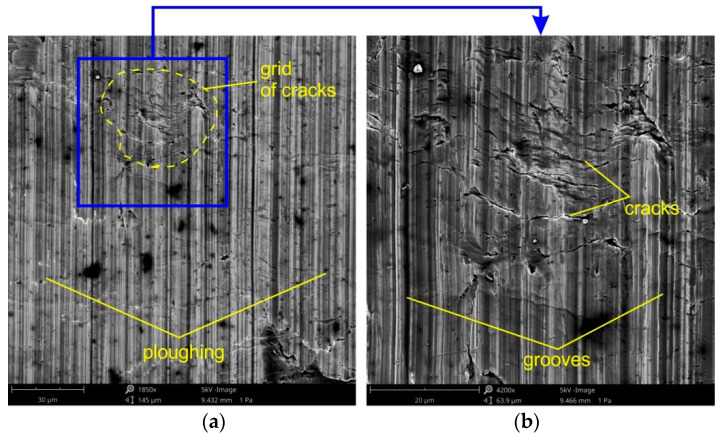
The specimen surfaces tested in dry friction (Ra = 0.63 μm, *h* = 18 mm, *w* = 14 mm, orientation 0°) at different magnifications: (**a**) ×1850 and (**b**) ×4200.

Under conditions of lubricating the sheet surface (Figure 18 and Figure 19) and during dry friction (Figure 19 and Figure 20), at a drawbead height of 6–12 mm, the surface after friction was characterized by a mixed proportion of flattened regions and valleys. The low roughness of the tools results in a large contact surface and the intensification of wear on the sheet surface at the level of surface asperities. In contrary, the high roughness of the tool provides less real contact surface but increases the contribution of the ploughing mechanism (Figure 21 and Figure 22). On the surfaces of the sheets tested with countersamples with a roughness of Ra = 0.63 and 1.25 mm, deep scratches with wear products are visible (Figure 21).

The surface layer of the tested sheets while passing through the drawbead with a height of *h* = 18 mm in dry friction conditions is characterized by a strong strain hardening, however, the degree of deformation of individual micro-areas in the sheet surface is quite varied. It proves the occurrence of different unit pressures in individual micro-contact areas. In regions that initially transferred load, together with repeated bending, unbending and reverse bending of the sheet strip material, the greatest strengthening is achieved, which reduces the formability of the material. At the same time, as a result of high pressure, the phenomenon of adhesion increases. This may create a grid of cracks (Figure 20).

### 3.6. Artificial Neural Networks

**Figure 23:**
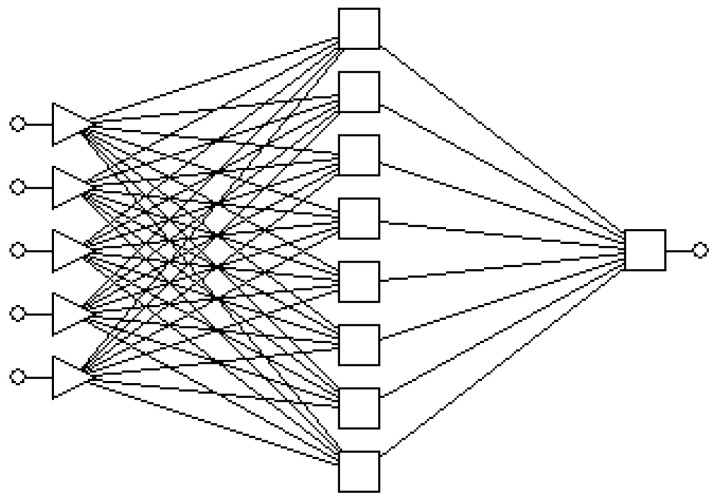
Multilayer ANN 5:5-8-1:1.

The Intelligent Problem Solver module built in the SSNs program is used to determine the appropriate structure of the neural network. This module enables the construction and evaluation of a large number of neural networks with a different structure of the hidden layer. The highest quality was characterized by the 5:5-8-1:1 network with 8 neurons in the hidden layer (Figure 23).

**Figure 24:**
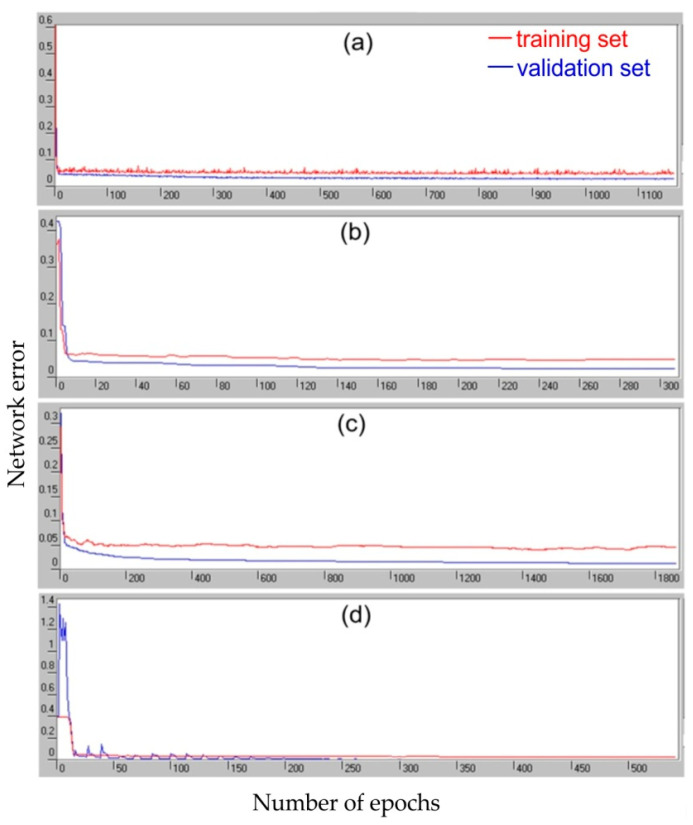
Variation of network errors during the training process with the algorithm: (**a**) back propagation, (**b**) conjugate gradients, (**c**) quasi-Newton and (**d**) Levenberg–Marquardt.

The training process carried out with the use of four algorithms was characterized by a continuous decrease in the RMS error of the training and validation set (Figure 24). The most effective algorithm for quickly reaching the minimum network error is the conjugate gradients algorithm (Figure 24b), which only required about 300 learning epochs. In contrast, the quasi-Newton algorithm took about 1900 epochs to reach the minimum value of the network response error (Figure 24c). It is worth noting that, in the whole training phase, the RMS error for the validation set was greater than the RMS error for the training set. 

From the point of view of network quality, the lowest RMS error value for the training set is characteristic for the network trained with the quasi-Newton algorithm (Table 3). The network trained with the Levenberg–Marquardt algorithm is characterized by a slightly greater error, and the largest error for both data sets was shown by the network trained with the back propagation algorithm.

Special attention should be paid to the Standard Deviation (SD) ratio and the correlation coefficient R^2^ [82]. The most important regression statistics of the training set and the validation set are presented in Table 4. The correlation coefficient of COF values presented at the network output during the training process and the values obtained as a result of the operating the neural network trained with the quasi-Newton method was approximately 0.996 (Table 4). This is the highest R^2^-value for the training set among all the training algorithms used. At the same time, for this data set, the SD ratio was the smallest and equalled about 0.0813. 

**Figure 25:**
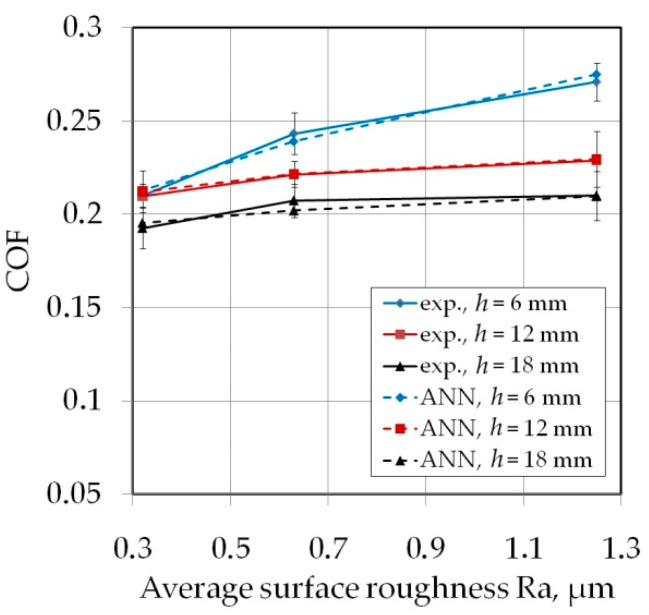
The effect of average surface roughness Ra of countersamples on the value of COF (orientation 0°, sample width *w* = 20 mm, dry friction conditions).

**Figure 26:**
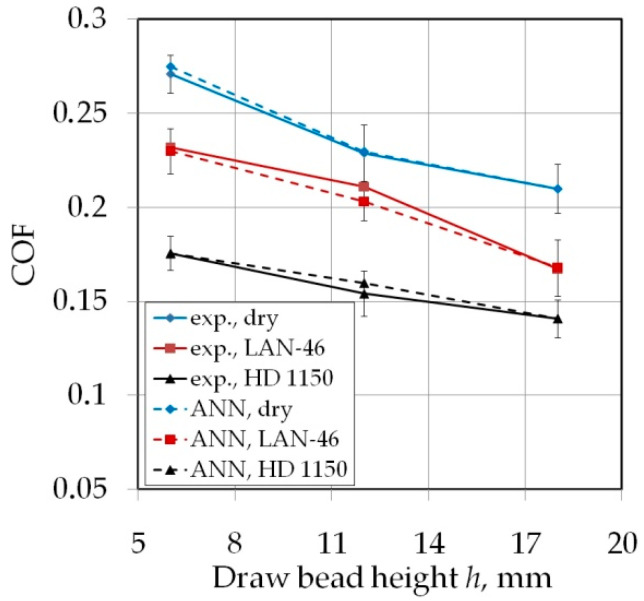
The effect of drawbead height on the value of COF (orientation 0, sample width *w* = 20 mm, average surface roughness of countersamples Ra = 1.25 μm).

Good regression properties of the ANN are reflected in a very good fit of coefficients of friction determined by the neural network to the experimental data represented by the training set (Figure 25 and Figure 26). The ability of the network to adapt to the training data having a clearly non-linear course is visible.

**Figure 27:**
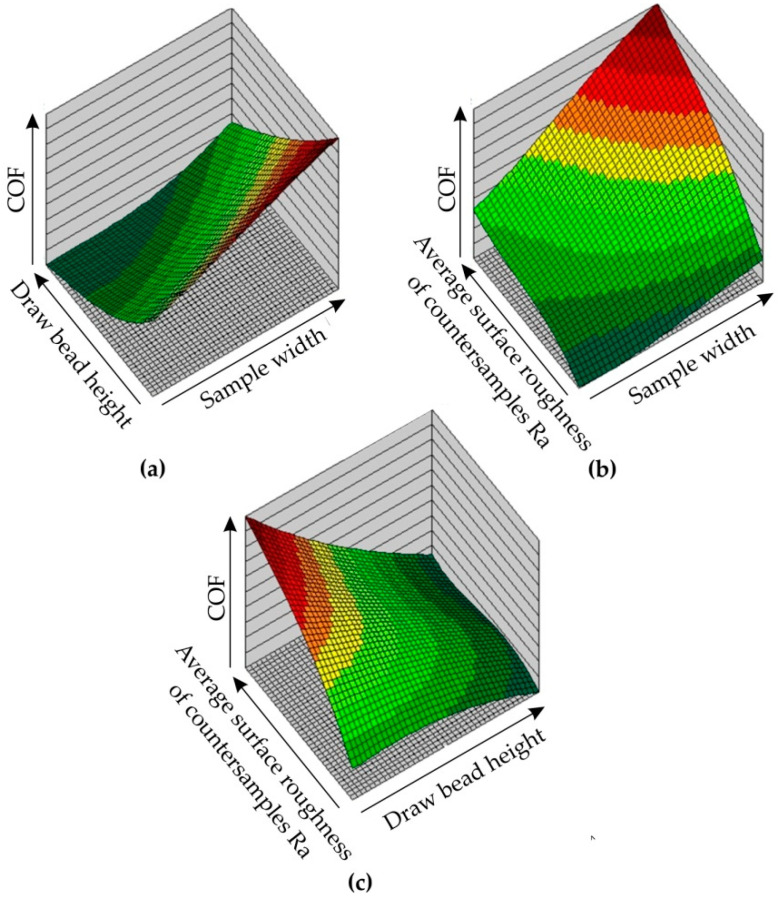
Response surfaces of 5:5-8-1:1 ANN showing the effect of (**a**) sample width and drawbead height, (**b**) sample width and average surface roughness of countersamples Ra, and (**c**) drawbead height and average surface roughness of countersamples Ra on the value of COF.

Figure 27 shows the response surfaces for the 5:5-9-1:1 ANN model, at different combinations of input parameters. The analysis of these surfaces allows the following conclusions to be drawn:

- The greater the drawbead height, the smaller the value of the COF (Figure 27a);
- An increase in the width of the sample leads to an increase in the value of the COF (Figure 27b);
- The greater the width of the sample, the greater the increase in the value of the COF (Figure 27b);
- Increasing the average surface roughness of countersamples increases the value of the COF, at low drawbead height values the increase is very fast, while the higher the drawbead height, the more equal the COF values are (Figure 27c).

The conclusions obtained on the basis of response surfaces are in line with the conclusions obtained on the basis of experimental research. The condition for a correct prediction of ANN is that the values of signals on the basis of which the network predicts the value of the COF fall within the range of parameters used to train the network. The neural network is able to extrapolate the dependence of the process parameters and the value of the COF beyond the range of the training data, however, the prediction error may increase significantly. The improvement of the prediction can be obtained by extending the training set.

The advantage of the model of the neural network applied is the possibility of determining non-linear relationships between many parameters of the friction process and the COF value. At the same time, it is not necessary to know the nature of the relationships between the network inputs and the predicted results [83,84]. A multilayer perceptron with an appropriate number of layers and neurons can model functions of any complexity [85]. A neural network is a black box in the sense that while it can approximate any function, studying its structure will not give you any insights into the structure of the function being approximated.

The application of the approach for predicting the technological parameters of the process using neural network methods is a modern method. Nevertheless, this approach only helps to find answers to local research problems. This method does not reveal the physical features of the process and does not allow the use of the results of the study for similar processes.

The limitation of the ANN model is that it only has the ability to model changes in the friction coefficient in the range of input parameter change values. Moreover, the accuracy of the ANN model prediction depends primarily on the data set that was taken into account during the training process. It should also be noted that too extensive a structure of the neural network used to model a given problem may lead to its overfitting, and thus, loss of the ability to generalise data. Each change of the training database, increasing the training set for example, requires additional training of the network or the creation of a new network taking into account the entire training set. The network structure must be re-selected to ensure the minimum value of network error. Moreover, the training process must be repeated. More data does not mean that a given network will have better predictive capabilities. It depends on whether the data will be determined or noisy. Of course, as long as possible, training set is desirable. However, the training data should evenly cover the range of values of input parameters. If the amount of training vectors is sufficient, several parameters can be set at the network output.

## 4. Conclusions

The conducted experimental studies and ANN modelling of COF of DC04 steel sheets in the drawbead region in SMF allow the following conclusions to be drawn:

- The width of the sheet strip tested in the drawbead simulator determines its behaviour during deformation, and thus, the real contact area of the sheet with countersamples. An increase in the width of the sample leads to an increase in the value of the COF.
- The chlorine-based HD 1150 compound was more effective in reducing COF than LAN-46 machine oil.
- Increasing the surface roughness of the countersamples reduces lubrication efficiency.
- The tests with the highest analysed drawbead height (*h* = 18 mm) did not show any significant influence of height on lubrication efficiency of the LAN-46 machine oil.
- Although the values of the COF for the two analysed sample orientations did not differ by more than 0.025, in most of the analysed cases, the COF value was higher for the sample orientation 90°.
- Analysis of the specimen surfaces after friction tests revealed that the main friction mechanisms while testing DC04 steel sheets are flattening, roughening and adhesion. The surface layer of the tested sheets while passing through the drawbead with the height *h* = 18 mm in dry friction conditions is characterized by severe adhesion, which leads to a grid of cracks.
- The most effective algorithm for the ANN training process was the quasi-Newton algorithm. The correlation coefficient of COF values presented at the network output during the training process and the values obtained as a result of the operation of the network 5:5-8-1:1 trained with this algorithm was approximately 0.996.
- Conclusions made on the basis of the response surfaces of ANN are in good agreement with the experimental results.

## Figures and Tables

**Table 1:** Basic mechanical parameters of the DC04 sheet metal (±standard deviation).

Specimen Orientation	Yield Stress *R_p02_*, MPa	Uniaxial Tensile Stress *R_m_*, MPa	Elongation *A_50_*, %	Strengthening Coefficient *K,* MPa	Strain Hardening Exponent *n*
0°	184.5 ± 3.0	303.9 ± 6.2	23.0 ± 0.6	490.4 ± 5.8	0.205 ± 0.003
90°	176.1 ± 0.5	296.0 ± 0.7	22.8 ± 0.3	465.7 ± 3.9	0.169 ± 0.002

**Table 2:** Basic surface roughness parameters of the DC04 sheet.

Sa, μm	Sq, μm	Sp, μm	Sv, μm	Sz, μm	Sal, mm	Str	Sdq	Ssk	Sku
1.32	1.54	10.48	10.31	20.79	0.05	0.93	0.15	−0.13	2.11

**Table 3:** Values of RMS error for training (T) and validation (V) sets.

Back Propagation Algorithm		Conjugate Gradients Algorithm		Quasi-Newton Algorithm		Levengerg-Marquardt Algorithm	
T	V	T	V	T	V	T	V
0.0316	0.0576	0.0286	0.0531	0.0158	0.0499	0.0195	0.0437

**Table 4:** Basic regression statistics for training (T) and validation (V) sets.

Parameter	Back Propagation Algorithm		Conjugate Gradients Algorithm		Quasi-Newton Algorithm		Levenberg–Marquardt Algorithm	
	T	V	T	V	T	V	T	V
Data mean	0.4553	0.4235	0.4553	0.4235	0.4553	0.4235	0.4553	0.4235
Data SD	0.1961	0.1981	0.1961	0.1981	0.1961	0.1981	0.1961	0.1981
Error mean	−0.0003	0.006	8.7 × 10^−5^	0.0054	4.5 × 10^−5^	0.0129	3.08 × 10^−6^	0.0032
Error SD	0.0318	0.0537	0.0287	0.0536	0.0159	0.0490	0.0195	0.0443
Abs error mean	0.0249	0.0415	0.0227	0.0419	0.0123	0.0404	0.0159	0.0361
SD ratio	0.1621	0.2710	0.1465	0.2708	0.0813	0.2473	0.0998	0.2238
Correlation	0.9867	0.9651	0.9892	0.9660	0.9966	0.9717	0.9950	0.9760

## Data Availability

The data presented in this study are available on request from the corresponding author.
